# Compressed Sensing Techniques Applied to Ultrasonic Imaging of Cargo Containers

**DOI:** 10.3390/s17010162

**Published:** 2017-01-15

**Authors:** Yuri Álvarez López, José Ángel Martínez Lorenzo

**Affiliations:** 1 Área de Teoría de la Señal y Comunicaciones, Universidad de Oviedo, Gijón (Asturias) 33203, Spain; alvarezyuri@uniovi.es; 2 Departments of Mechanical & Industrial Engineering and Electrical & Computer Engineering, Northeastern University, Boston, MA 02115, USA

**Keywords:** non-destructive testing, ultrasound imaging, cargo inspection, angle beam transducers, Lamb waves, compressed sensing

## Abstract

One of the key issues in the fight against the smuggling of goods has been the development of scanners for cargo inspection. X-ray-based radiographic system scanners are the most developed sensing modality. However, they are costly and use bulky sources that emit hazardous, ionizing radiation. Aiming to improve the probability of threat detection, an ultrasonic-based technique, capable of detecting the footprint of metallic containers or compartments concealed within the metallic structure of the inspected cargo, has been proposed. The system consists of an array of acoustic transceivers that is attached to the metallic structure-under-inspection, creating a guided acoustic Lamb wave. Reflections due to discontinuities are detected in the images, provided by an imaging algorithm. Taking into consideration that the majority of those images are sparse, this contribution analyzes the application of Compressed Sensing (CS) techniques in order to reduce the amount of measurements needed, thus achieving faster scanning, without compromising the detection capabilities of the system. A parametric study of the image quality, as a function of the samples needed in spatial and frequency domains, is presented, as well as the dependence on the sampling pattern. For this purpose, realistic cargo inspection scenarios have been simulated.

## 1. Introduction

One of the measures taken for protecting against the smuggling and illegal movement of people across borders, is the use of scanners for cargo inspection [1,2]. X-ray-based radiographic systems are the most commonly used apparatus at checkpoint facilities as they are based on a mature technology, capable of providing high resolution images [3,4]. However, they emit ionizing radiation which is hazardous for people; moreover, their cost and size restrict the places from which they can be deployed. Millimeter-wave scanners, emitting non-ionizing radiation, have been found to be effective in personnel screening [5]. However, electromagnetic waves cannot penetrate metallic walls, so this technology is not valid for the inspection of metallic cargo containers.

As explained in [6], compartments can be camouflaged within the metallic structure of the cargo, in order to conceal radioactive materials, as metals with a high atomic Z number (e.g., lead, Z = 82) diminish their radioactive signature, thus reducing the probability of detection using gamma-ray spectrum analysis and/or neutron detectors.

To improve the detection capabilities of the aforementioned systems, ultrasonic imaging has been proposed in [6,7]. The goal is to detect the footprint of metallic containers, as well as potentially smuggled goods, concealed within the metallic structure of the cargo. The system consists of an array of acoustic transceivers that is attached to the metallic structure of the cargo, thus exciting an acoustic Lamb wave [8,9]. Gaps, joints, or any other metal discontinuity, create reflections that are recorded by the transceivers. Finally, a backpropagation imaging algorithm [6,10] is applied in order to recover an acoustic image revealing the placement of these discontinuities.

**Figure 1:**
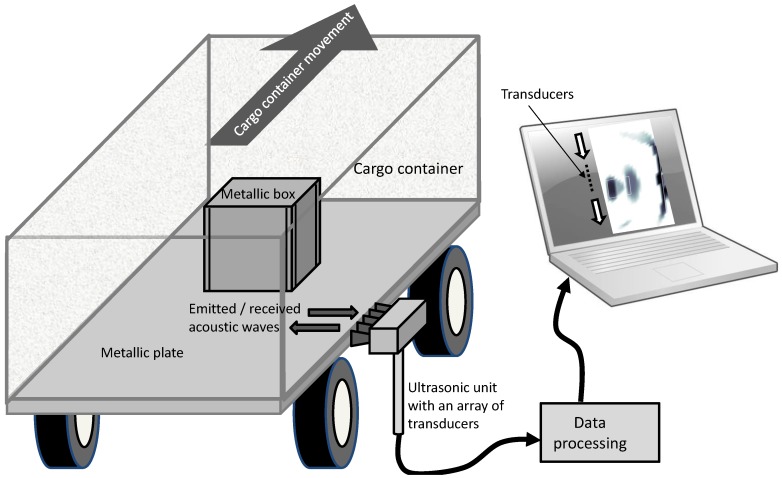
Proposed layout for ultrasound imaging-based cargo inspection.

An overview of the proposed ultrasound imaging system for cargo inspection is depicted in Figure 1. The size of the array of transducers limits the region of the cargo that can be scanned. Thus, the movement of the cargo when passing through the inspection facility is used to create the full ultrasound image. However, as discussed in [6], the size of the array of transducers impacts the resolution of the final ultrasound image; increasing the number of transducers improves the image quality, but also makes the scanning system more expensive.

In the majority of cases, the kind of ultrasound images produced from the inspected cargo are likely to be sparse, as Lamb wave reflections will occur where discontinuities in the metallic structure of the cargo are detected. Taking into account this feature, it is possible to reduce the number of measurements needed to recover the ultrasound image, without jeopardizing the probability of detection. In this sense, Compressed Sensing (CS) techniques have proved to be quite efficient methods for recovering a sparse signal from a subsampled set of measurements.

In the field of ultrasound imaging, CS has been successfully applied in [11,12,13,14] during medical imaging, and in [15,16,17,18,19] during non-destructive testing. In all cases, CS is applied in order to reduce the number of samples required to recover an ultrasound signal [12,13,15,17] or ultrasound image [16,18,19]. The different approaches proposed in these works will be discussed in Section 3.

From a hardware implementation point-of-view, CS can contribute towards reducing the number of transducers of the acquisition system, thus reducing complexity and cost [13,16]. Following these strategies, this study discusses the applicability of CS techniques to the ultrasound imaging system for the cargo inspection described in [6], aiming to reduce the number of acoustic transducers while maintaining the detection capabilities. For this purpose, two sampling patters are presented, and the feasibility of each patter’s practical implementation is discussed. In addition to this, the impact of spatial and frequency subsampling in the reconstructed CS images is analyzed from a quantitative point-of-view, in order to identify the minimum number of transducers required, without degrading the detection capabilities of the system. The achieved performance will be compared with the state-of-the-art system in Section 5.

## 2. Backpropagation Imaging Algorithm

This section describes the basic backpropagation algorithm [6,10] used to retrieve the ultrasound image. The acoustic pressure *p*(*x*, *y* = 0, *f*) is recorded in a set of measurement positions (*Nx*) along the *x* axis (*y* = 0), e.g., by using an array of transducers. For each measurement position, the acoustic pressure will exhibit a frequency response, where the highest measured frequency, *fmax*, and the number of samples, *Nf*, is limited by the specifications of the transducer.

Once the spatial and frequency response is stored, the ultrasound image reflectivity *ρ* can be calculated by simply backpropagating the recorded acoustic pressure *p*(*x*, *y =* 0, *f*) over a frequency range *f,* and spatial acquisition domain *x*, to the imaging domain (*x*’, *y*’):

(1) $$\rho \left(x^{\prime },y^{\prime }\right)=\overset{f_{max}}{\underset{f=0}{\sum }}\overset{x_{N}}{\underset{x=x_{1}}{\sum }}p\left(x,y=0,f\right)e^{+j4\pi Rf/c_{med}}$$

where *R* is the Euclidean distance between the n-th acquisition point (*x*, *y* = 0) and the evaluation point (*x*’, *y*’), $R={\left({\left(x-x^{\prime }\right)}^{2}+{\left(y^{\prime }\right)}^{2}\right)}^{1/2}$, and *c*_med_ is the acoustic wave propagation speed in the medium of either longitudinal p-wave, or transversal s-wave. Equation 1 can be expressed in a matrix form:

(2) $${\left(\rho \right)}_{M\times 1}={\left[{\left(S\right)}_{N\times M}\right]}^{+}{\left(p\right)}_{N\times 1}$$

${\left[S\right]}^{+}$ denotes the pseudoinverse of S. *M* is the number of points (image pixels) where the reflectivity *ρ* is recovered; *N* is the overall number of measurements, *N* = *Nf* × *Nx*, samples; *p* denotes all the measurements, i.e., the frequency response of the acoustic pressure in all the measurement positions. (S)*_N_*_×*M*_ is the sensing matrix, where each element *s* for the *m*-th image pixel and the *n*-th observation point and frequency, is defined as:

(3) $$s\left(m,n\right)=e^{-j4\pi R\left(x_{n};x_{m}^{\prime },y_{m}^{\prime }\right)f_{n}/c_{med}}$$

*m* ranging from 1 to *M*, and *n* ranging from 1 to *N* = *Nf* × *Nx*. Vectors *f* and *x* have to be redefined so that they have the same number of elements as *p*. For this purpose, they are constructed as follows:

(4) $${\left(f\right)}_{1\times N}=\underset{N_{f}}{\underset{︸}{\left(\begin{array}{cc} \begin{array}{cc} \underset{N_{x}}{\underset{︸}{\left(\begin{array}{ccc} f_{1} & \cdots & f_{1} \end{array}\right)}} & \underset{N_{x}}{\underset{︸}{\left(\begin{array}{ccc} f_{2} & \cdots & f_{2} \end{array}\right)}} \end{array} & \begin{array}{cc} \ldots & \underset{N_{x}}{\underset{︸}{\left(\begin{array}{ccc} f_{N_{f}} & \cdots & f_{N_{f}} \end{array}\right)}} \end{array} \end{array}\right)}}$$

(5) $${\left(x\right)}_{1\times N}=\underset{N_{f}}{\underset{︸}{\left(\begin{array}{cc} \begin{array}{cc} \underset{N_{x}}{\underset{︸}{\left(\begin{array}{ccc} x_{1} & \cdots & x_{N_{x}} \end{array}\right)}} & \underset{N_{x}}{\underset{︸}{\left(\begin{array}{ccc} x_{1} & \cdots & x_{N_{x}} \end{array}\right)}} \end{array} & \begin{array}{cc} \ldots & \underset{N_{x}}{\underset{︸}{\left(\begin{array}{ccc} x_{1} & \cdots & x_{N_{x}} \end{array}\right)}} \end{array} \end{array}\right)}}$$

where the elements of vectors *f* and *x* are used to construct the elements *s* of matrix S.

Thus, it can be stated that each acoustic pressure sample, *p*(*x*, *y* = 0, *f*), can be expressed as a linear combination of *M* bases *S*:*p* = *Sρ*.

The backpropagation imaging algorithm described in this section can be implemented using the Fourier transform, as described in [6]. Firstly, the acoustic pressure *p*(*x*, *y =* 0, *f*) is transformed to the spectral domain in both frequency and space, *P*(*k*_x_, *k_y_*), where *k*_x_ is the spatial frequency, *k_y_* = (*k*_med_^2^ − *k*_x_^2^)^1/2^, *k*_med_ is the wavenumber, and *k*_med_ = 2π*f*/c_med_. Spectral domain representation allows the identification of plane wave contributions, thus enabling the filtering of non-desired plane wave contributions (e.g., reflections from the edges/sides of the object-under-inspection) [20]. Following this, the filtered acoustic pressure in the spectral domain *P*_filtered_(*k*_x_, *k_y_*) is displaced back to the spatial domain *p*_filtered_(*x*, *y =* 0, *f*), by means of an inverse Fourier transform. Finally, Equation (1) is applied in order to recover the ultrasound image reflectivity *ρ* in the imaging domain (*x*’, *y*’).

The use of Fast Fourier Transform (FFT) requires the samples to be uniformly spaced (in both the space and time axes). This highlights a limitation concerning CS applicability, which will be justified in Section 3. Therefore, CS will be applied over the backpropagation imaging algorithm described in Section 2.

## 3. Compressed Sensing Technique

According to CS theory [21,22,23,24], sparse signals can be recovered from samples taken at a lower sampling rate than Nyquist criterion; e.g., the spatial domain samples can be spaced at more than *λ_min_*/2 = *c*_m_/(2*f_max_*).

As stated in Section 2, the acoustic pressure *p* is expressed as a linear combination of *M* bases. This signal *p* is said to be $M_{\neq 0}$-sparse if it is a linear combination of only $M_{\neq 0}$ basis vectors. This means that ${\left(\rho \right)}_{M\times 1}$ only has $M_{\neq 0}$ non-zero elements, with $M_{\neq 0}\ll M$.

Different strategies have been developed to make signals sparser. Some of these are based on signal expansion in a set of basis functions, where signal coefficients are sparse. In this sense, Fourier bases and Wave Atoms are proposed in [13,16]. It must be noted that the degree of sparsity achieved with these transformations depends on the nature of the signal.

Thus, provided that *ρ* is sparse, CS theory states than it can be recovered from a subset of *p*, denoted $p_{sub}={\left(p_{sub}\right)}_{N_{sub}\times 1}$. A projection matrix relates *p* and *p_sub_*:

(6) $${\left(p_{sub}\right)}_{N_{sub}\times 1}={\left(\Phi \right)}_{N_{sub}\times N}{\left(p\right)}_{N\times 1}$$

Thus, Equation (2) can be rewritten in terms of Equation (6) as:

(7) $${\left(p_{sub}\right)}_{N_{sub}\times 1}={\left(\Phi \right)}_{N_{sub}\times N}{\left(p\right)}_{N\times 1}={\left(\Phi \right)}_{N_{sub}\times N}{\left(S\right)}_{N\times M}{\left(\rho \right)}_{M\times 1}={\left(\Theta \right)}_{N_{sub}\times M}{\left(\rho \right)}_{M\times 1}$$

where Θ= ΦS. Equation (7) is underdetermined, as *N_sub_* << *M*. However, if *ρ* is sparse, and Θ obeys a Restricted Isometry Property (RIP), Equation (7) can be solved by means of convex programming. RIP is satisfied if the sensing matrix Φ and the orthonormal bases *S* are incoherent, a condition that has been proved to be widely fulfilled if the sampling operator Φ is a random matrix. Examples presented in this contribution will discuss the trade-off between a feasible realization of the sensing matrix Φ, and the randomness degree.

*ρ* can be reconstructed by solving a constrained optimization problem:

(8) $$min{∥{\left(\rho \right)}_{M\times 1}∥}_{1}\;\mathrm{subject}\;\mathrm{to}\;{\left(p_{sub}\right)}_{N_{sub}\times 1}={\left(\Theta \right)}_{N_{sub}\times M}{\left(\rho \right)}_{M\times 1}$$

where ${∥\;∥}_{1}$ is the L-1 norm. Measurements are typically contaminated with noise, so the minimization problem is changed to:

(9) $$min{∥{\left(\rho \right)}_{M\times 1}∥}_{1}\;\mathrm{subject}\;\mathrm{to}\;{∥{\left(p_{sub}\right)}_{N_{sub}\times 1}={\left(\Theta \right)}_{N_{sub}\times M}{\left(\rho \right)}_{M\times 1}∥}_{2}<\varepsilon$$

The value of the regularization parameter, *ε*, is set by means of a cross-validation technique [23]. Different solvers have been considered for Equation (9) minimization, such as the Spectral Projected Gradient for L1 minimization (SPGL1) [25], or Nesta [26]. As explained in [27], optimal recovery of *ρ* from subsampled *p*, *p_sub_*, occurs when the number of non-zero elements of *ρ* (i.e., image pixels with non-zero reflectivity values) is around $N_{sub}\approx 4M_{\neq 0}$.

For the problem analyzed in this contribution, ultrasound images are already quite sparse (some examples are also shown in [6]), as the ultrasound image amplitude is different from zero, only in those points (*x*’, *y*’) (or pixels) corresponding to the reflections of the guided ultrasonic waves (i.e., the footprint of the detected objects). For this kind of sparse imagery, Total Variation (TV) minimization [28] has been found to be quite suitable, as it minimizes ∇ρ (Equation (10)), making the image sparser than ρ. Note that, if ρ is the acoustic reflectivity image, with ρ > 0 being the footprints of the detected objects, then ∇ρ are the footprint contours.

(10) $$min{∥\nabla {\left(\rho \right)}_{M\times 1}∥}_{1}\;\mathrm{subject}\;\mathrm{to}\;{∥{\left(p_{sub}\right)}_{N_{sub}\times 1}={\left(\Theta \right)}_{N_{sub}\times M}{\left(\rho \right)}_{M\times 1}∥}_{2}<\varepsilon$$

CS cannot be applied with the Fourier-based filtering technique introduced in [6], because of the random nature requirement of the sensing matrix Φ that allows Θ= ΦS to fulfill the RIP. If evenly-spaced samples are taken in both time and spatial domains, then the coherence of Θ= ΦS, defined as μ($\Theta^{*}$, Θ) [16,29], will be higher than for the cases in which random sensing matrices Φ are constructed. As proven in [29], when focusing on millimeter-wave imaging applications, random sampling schemes result in lower coherence values, which thus results in accurate object-under-test profile reconstruction with a reduced set of points (25% of the points required by Nyquist criterion).

## 4. Application Example

### 4.1. Description of the Example

**Figure 2:**
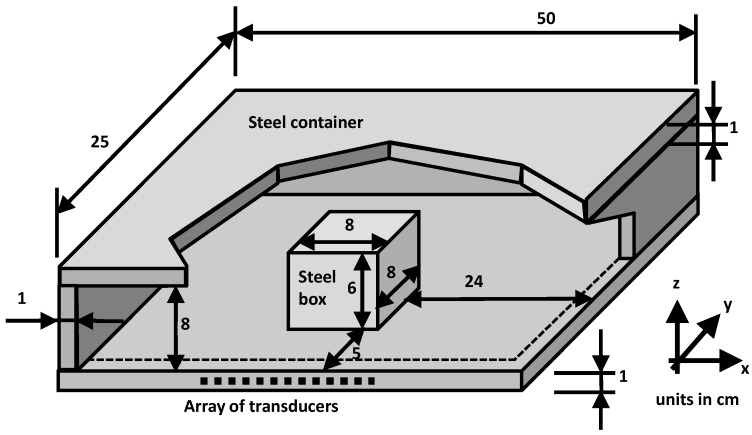
Problem-under-test: steel container with a metallic box in it. An array of transducers is placed on one side of the bottom plate of the container.

An evaluation of the feasibility of CS for ultrasound imaging is now presented. For this purpose, a realistic example, depicted in Figure 2, has been considered. The scenario consists of a container made of 1 cm thick steel plates, with a steel box inside it. This example is of particular interest to cargo inspection, as high energy radiation (X-ray) is required to penetrate not only the cargo container, but also the metallic box inside it [6].

The scenario is simulated using a 3D finite-element method (FEM) [30], considering the following parameters for steel: a P-wave velocity of 5960 m/s, a S-wave velocity of 3220 m/s, and a density of 8000 kg/m^3^. Both the simulation and the ultrasound imaging technique have been run using a conventional laptop with 4 GB RAM and Intel^®^ Core™ i5 Quad-core CPU at 2.67 GHz. Due to the computational limitations of simulating a real size container, which may range from 2 to 10 m, this example is a scaled version. Only the thickness of the metallic plates have been kept the same as in a real scenario.

An array of *Nx* = 18 ultrasound transducers, evenly spaced at 1 cm intervals from *x* = 14 cm to *x* = 31 cm, is attached to one of the edges of the container’s metallic base plate (which corresponds to layout I in Figure 5 of [6]). A *f* = 100 kHz windowed toner burst has been chosen as the excitation signal. For this frequency and plate thickness (1 cm), only the S_0_ Lamb mode is excited, so dispersion is kept low, as no higher dispersive modes are excited (Figure 6 and Figure 7 of [6]).

Note that, as opposed to the examples presented in [6], the array of transducers is placed far from the corners of the base plate. As explained in Section 3, CS formulation prevents the use of Fourier-based ultrasound imaging for filtering out non-desired contributions, mainly due to excited edge plate modes. Thus, to avoid additional image degradation, the array of transducers is placed close to the center of the base plate edge, minimizing the distortion effects of edge plate modes.

The recorded acoustic pressure in the transducers is evenly sampled from 0 Hz to *fmax* = 100 kHz, with *Nf* = 42, resulting in *N* = *Nf* × *Nx* = 42 × 18 = 756 samples. The ultrasound image is recovered in a 0.5 m × 0.5 m domain, discretized in *M* = 2601 pixels (∆*x*’ = ∆*y*’ = 1 cm).

### 4.2. Analysis of the Sampling Schemes

As stated in Section 1, the goal of this work is to assess the CS applicability in order to decrease the number of acoustic pressure samples, *N*, while maintaining the quality of the ultrasound image, *ρ*, which determines the detection capability of the scanning system. In order to achieve this goal, an exhaustive analysis of different sampling patters is presented in this section.

**Figure 3:**
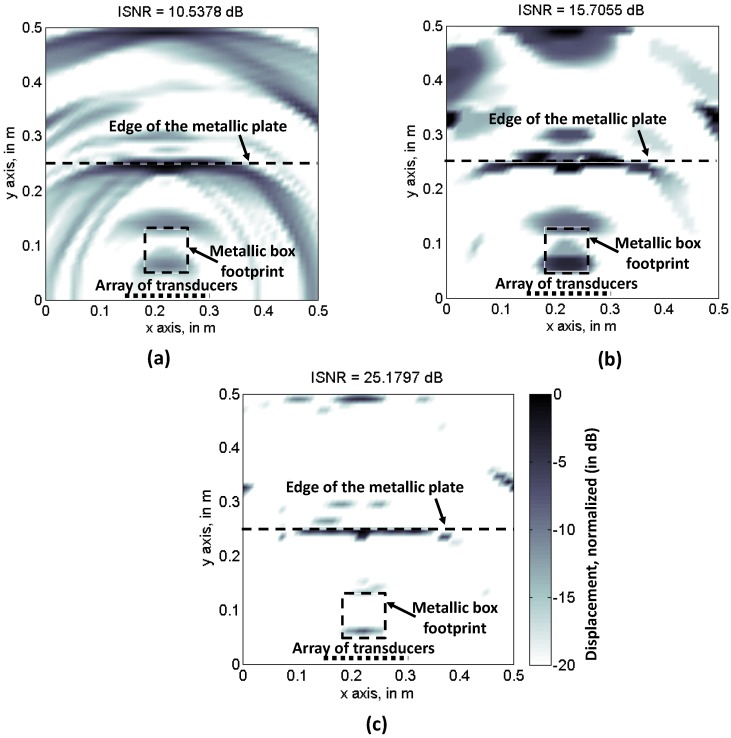
Recovered reflectivity when all the frequencies and spatial positions are considered. (**a**) Backpropagation imaging; (**b**) Compressed Sensing, Total Variation; (**c**) Compressed Sensing, SPGL1.

First, backpropagation imaging and CS algorithms are applied using the entire set of acoustic pressure samples (*N* = 756). Results for backpropagation imaging are depicted in Figure 3a. Note that the footprint of the metallic box, as well as the reflection occurring in the far edge of the metallic base plate. As Fourier imaging cannot be applied to filter out non-desired contributions, a degradation of the image sharpness, with respect to the imaging results presented in [6], can be noticed, especially in the areas close to the left and right sides of the plate. Quantitative assessment of the image quality is provided by the image signal-to-noise ratio (ISNR), defined in Equation (15) of [23], which is:

(11) $$\mathrm{ISNR}\;=\;10\log_{10}\left\{\frac{Q-Q_{R}}{Q_{R}}\frac{\sum_{m=1}^{Q_{R}}{\left|\mu_{m}\right|}^{2}}{\sum_{n=1}^{Q-Q_{R}}{\left|\mu_{n}\right|}^{2}}\right\}$$

where *Q* is the total number of pixels in the normalized image, *Q*_R_ are the pixels whose amplitude is greater than −10 dB, ${\left|\mu_{m}\right|}^{2}$ is the image pixel amplitude greater than −10 dB, and ${\left|\mu_{n}\right|}^{2}$ is the image pixel amplitude of the remaining *Q* − *Q*_R_ pixels [23].

CS results using SPGL1 (minimization of Equation (9)) and TV (minimization of Equation (10)) solvers are depicted in Figure 3b,c, respectively. TV ISNR is 5 dB higher than backpropagation image ISNR, while SPGL1 further improves ISNR 10 dB with respect to TV. However, it must be remarked that this parameter is valid for assessing the sharpness of an image, but not the accuracy of the imaging result.

**Figure 4:**
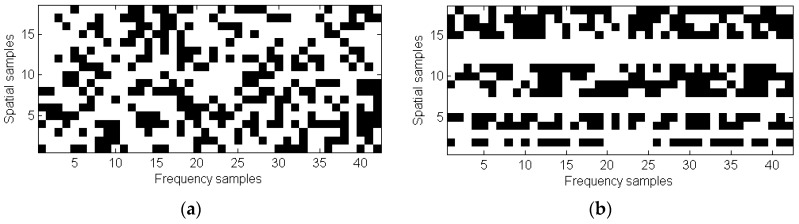
Sampling patterns for *N_sub_* = 0.6*Nx* × 0.6*Nf* = 0.36*N*. (**a**) Samples in frequency and spatial domain are randomly chosen; (**b**) Random sampling in frequency for a given random set of spatial positions. Black dots represent selected positions.

To fulfill the RIP, the sensing matrix Φ is generally a binary random matrix [12,13,14,15,16,17,18,19,20,21,22,23,24,25,26,27]. In this situation, *N_sub_* out of *N* positions arranged in a *Nx* × *Nf* matrix are selected. For example, Figure 4a represents a random sensing matrix that fulfills *N_sub_* = 0.6*Nx* × 0.6*Nf* = 0.36*N*. Note that although only 36% of the samples are selected, there is at least one selected spatial sample for each frequency, and vice-versa.

As discussed in [11,12,13,29], while random sensing matrices minimize the coherence of Θ= ΦS, it must be taken into account that physical limitations of the data acquisition system in which CS is going to be applied, may prevent random sampling from being advantageous, with respect to conventional sampling at a Nyquist rate. Considering this, Quinsac et al. [11] analyzes several sampling patterns, proposed as a trade-off between maximizing the incoherence of Θ (random sampling), and the practical implementation of the acquisition system (partial random sampling).

In a similar way to [11], the ultrasound system described in this contribution places every transducer along the *x*-axis and acquires the acoustic pressure *p* in a certain frequency band. From a hardware complexity point-of-view, the limiting factor is the number of transducers required, but not the number of frequency samples. Thus, the sampling pattern first requires a fixed random sequence of spatial sampling positions (i.e., transducers), and then, different random sequences of frequency samples can be generated for each spatial position. This feasible, and partially random, sampling scheme is depicted in Figure 4b, also for *N_sub_* = 0.6*Nx* × 0.6*Nf* = 0.36*N*.

Even though the number of selected samples in Figure 4a,b is the same, *N_sub_* = 0.36*N*, in the case of Figure 4a, there is at least one selected frequency sample for each spatial position, whereas in Figure 4b, only 60% of the spatial samples have frequency samples. Thus, the sampling scheme of Figure 4b results in an effective reduction of the number of transducers.

**Figure 5:**
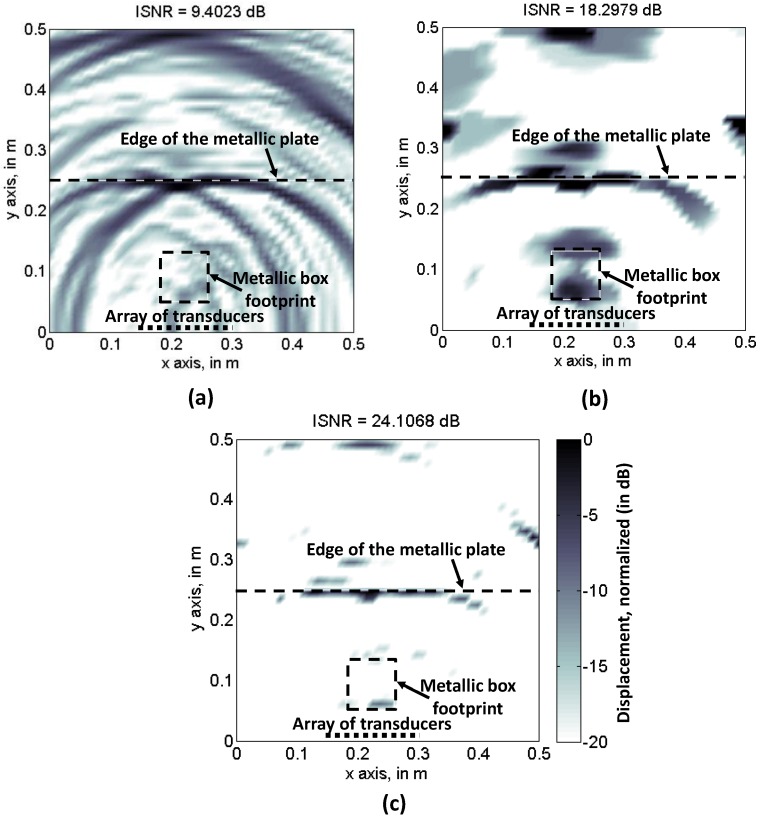
Recovered reflectivity from the spatial and frequency samples taken according to Figure 4a sampling scheme (random sampling). (**a**) Backpropagation imaging; (**b**) Compressed Sensing, Total Variation; (**c**) Compressed Sensing, SPGL-1.

CS performance for Figure 4 subsets is tested. Ultrasound imaging results are depicted in Figure 5b (TV) and Figure 5c (SPGL1), for the Figure 4a sampling pattern. Backpropagation is also applied using this reduced set of samples, and the results are shown in Figure 5a. As the Nyquist sampling rate is not fulfilled, the back-propagated ultrasound image is distorted due to aliasing. Note that the ISNR value is similar to Figure 3a, confirming that this parameter is only valid for image sharpness evaluation. In the case of CS-TV results, Figure 5b, partial degradation with respect to Figure 3b can be identified, although both the edge of the metallic plate, and the box footprint, are distinguishable. With respect to CS-SPGL1, Figure 5c, note that, even though the ISNR is again higher than the TV, only the edge of the metallic plate is clearly noticeable.

**Figure 6:**
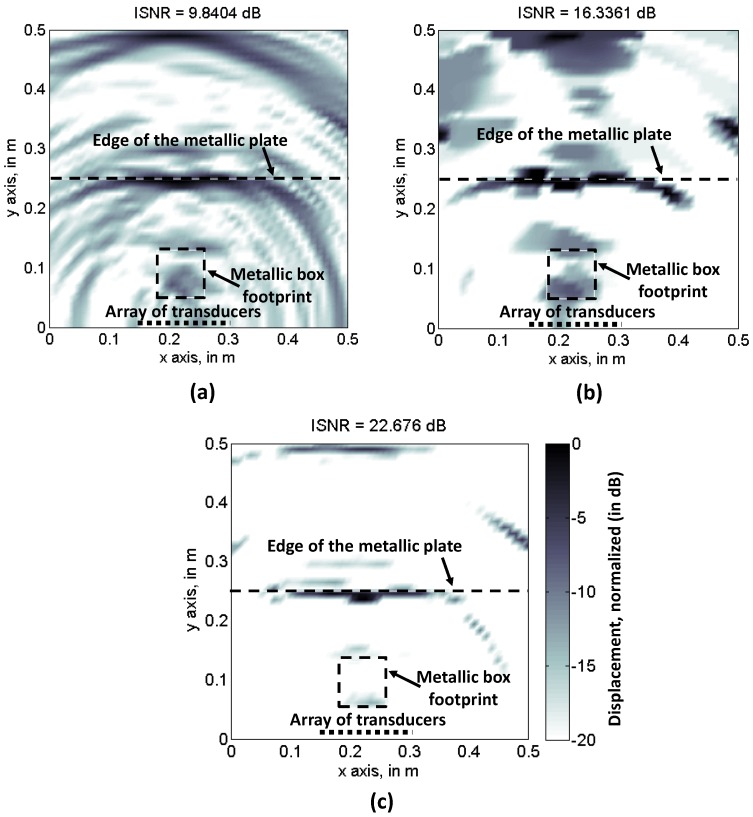
Recovered reflectivity from the spatial and frequency samples taken according to Figure 4b sampling scheme (partial random sampling). (**a**) Backpropagation imaging; (**b**) Compressed Sensing, Total Variation; (**c**) Compressed Sensing, SPGL-1.

Imaging results for the partially random sampling scheme depicted in Figure 4b are shown in Figure 6a–c, for backpropagation, CS-TV, and CS-SPGL1 imaging, respectively. In the case of Figure 6a, little improvement with respect to Figure 5a can be observed, as the sampling in the spatial domain (*x*-axis) is the same for all of the frequencies. Having said this, aliasing effects that degrade the image quality are present. The CS-TV image is slightly worse than Figure 5b because of the lower degree of randomness of the sensing matrix. The same applies to CS-SPGL1, Figure 5c, where the metallic box footprint can hardly be identified, despite the higher ISNR value of the reconstructed image. Thus, only CS-TV will be further considered.

Once the sampling schemes have been defined, the next step is to evaluate the accuracy of the reconstructed image for different sizes of subsampled sets, *N_sub_* = α*Nx* × β*Nf,* α and β being the percentage of samples. As the ISNR cannot be used to define a metric that quantifies the reconstruction accuracy, the method proposed in Section IV.B of [31] has been applied. The recovered CS ultrasound image is first converted into a binary bitmap and then compared with a mask that fits the metallic box footprint and the reflection, due to the opposite edge of the metallic plate. A CS image intensity threshold of −20 dB is selected for binary bitmap conversion. Following this, the binary image and the mask are compared, pixel by pixel. The metric defined to evaluate the quality of the CS image is:

(12) (Pcorr−Pwrong)/Pmask,

where *Pcorr* is the number of CS image pixels within the mask, *Pwrong* is the number of CS image pixels outside the mask, and *Pmask* is the number of pixels of the mask.

**Figure 7:**
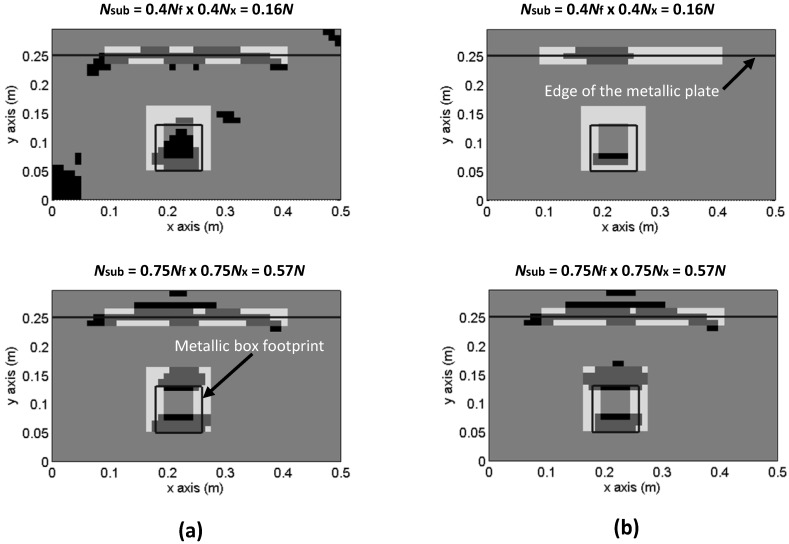
Evaluation of Compressed Sensing images quality. Pixelized CS-TV image and mask representing the metallic box footprint and the opposite edge of the metallic plate. (**a**) Results using Figure 4a sampling scheme (random sampling); (**b**) Results using Figure 4b sampling scheme (partial random sampling).

Several examples of the pixelized CS image, compared to the mask, are shown in Figure 7. Results for *N_sub_* = 0.4*Nx* × 0.4*Nf* = 0.16*N* and *N_sub_* = 0.75*Nx* × 0.75*Nf* = 0.57*N*, both with full random sensing matrix, are plotted in Figure 7a. Those based on the use of partial random sensing matrix are plotted in Figure 7b. No significant discrepancies are observed for *N_sub_* = 0.56*N*, whereas for *N_sub_* = 0.16*N,* random sampling outperforms partial random sampling.

**Figure 8:**
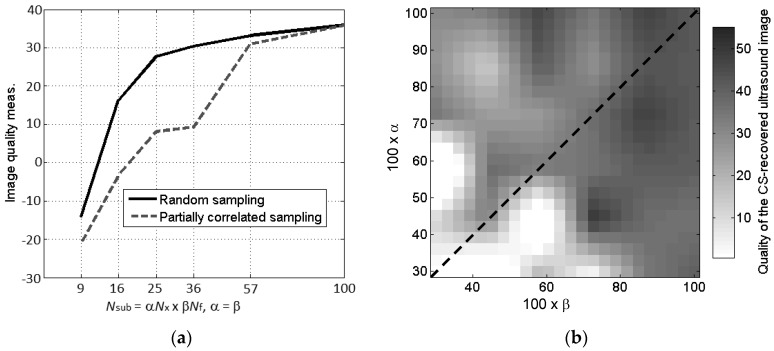
(**a**) Comparison of the CS-TV image quality defined by Equation (12) for the two sampling schemes; (**b**) Image quality analysis (0-white meaning bad performance, and 1-black meaning good performance) of the CS image quality for different number of frequency and spatial samples (partial random sampling). Dashed line denotes the same number of frequency and spatial samples.

Results of the quantitative analysis of CS image quality are depicted in Figure 8. Note that, due to the random nature of the sensing matrix, for each combination of *N_sub_* = α*Nx* × β*Nf*, 10 sampling matrices have been generated, before applying CS. The image quality is evaluated for the 10 resulting ultrasound images, and the average image quality value is stored.

From the results plotted in Figure 8, it is confirmed that full random sensing matrices allow for smaller subsets of samples than the partial random sampling scheme. However, practical implementation of the ultrasound-based imaging for cargo inspection requires the latter. Due to this, it is also of interest to analyze CS reconstruction accuracy for α ≠ β when the partial random sampling scheme is applied. In this case, α corresponds to the number of transducers considered, and β, to the number of frequency samples for each transducer. The results in Figure 8b show that the impact of the number of frequency and spatial samples in CS performance, when focusing on the CS image quality, is similar, illustrating a significant decrease in the image quality for *N_sub_* < [0.25, 0.35]*N*. Note that for a full random sensing matrix, CS image quality is maintained with as few as *N_sub_* = 0.5*Nx* × 0.5*Nf* = 0.25*N*.

## 5. Discussion

The application of CS techniques to the ultrasound-based imaging system used in cargo inspection [6,7] allows a reduction in the number of samples of 65%–75% with respect to the full set of acoustic pressure samples, when considering a partial random sampling scheme. Although this is slightly less efficient than a full random sampling scheme, it can be implemented to achieve the final goal of reducing the number of elements in the array of transducers.

**Figure 9:**
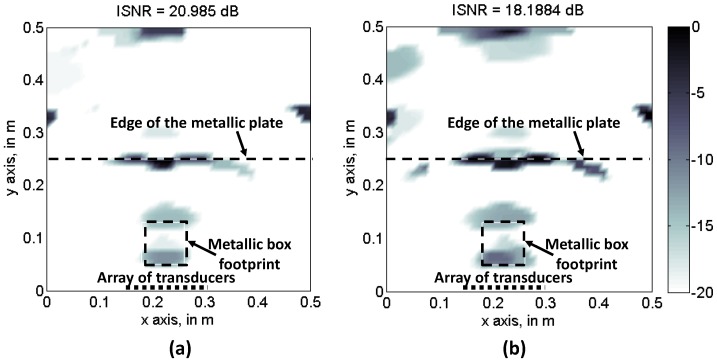
Recovered reflectivity, CS-TV with partial random sampling scheme. (**a**) *N_sub_* = 0.4*Nx* × 1*Nf* = 0.4*N*: 40% of transducers. For each selected transducer, all the frequency samples are considered; (**b**) *N_sub_* = 0.6*Nx* × 0.6*Nf* = 0.36*N*: 60% of transducers. For each selected transducers, 60% of the frequencies are considered.

Figure 8b also shows the influence of the number of frequency and space samples on the recovered CS image quality. In general, increasing the subsampling factor of one parameter requires a higher number of samples of the other, for a given image quality. Besides, when considering this problem, image quality seems to be more sensitive to frequency subsampling. The result of interest from Figure 8b is the fact that it is possible to reduce the number of spatial samples (i.e., the number of transducers), at the expense of increasing the number of frequency samples, as the latter has little impact on the overall complexity of the ultrasound imaging system. To illustrate this concept, Figure 9 compares the recovered reflectivity for 40% of the transducers with *N_sub_* = 0.4*Nx* × 1*Nf* = 0.4*N* (Figure 9a), and for 60% of the transducers with *N_sub_* = 0.6*Nx* × 0.6*Nf* = 0.36*N*. In both cases, the image quality is almost the same (also observed in Figure 8b), but in the former case, the number of transducers is only 40% of the full array.

If compared to the existing literature, similar performance in the reduction of the number of samples is achieved, i.e., 20%–30%. In [13], where CS is applied to reduce the number of sensing positions in a medical ultrasound imaging system, a maximum reconstruction error of 5.5 dB is obtained, with as few as 20% of the number of samples of the full array. Wagner et al. [14] proves that CS is able to recover the original signal with a probability of 90% and a subsampling factor of 5 (i.e., 20% of the full array of samples). And Di Ianni et al. [16] shows that an accurate recovery of the excited Lamb waves can be achieved with less than 34% of the measurements from the original grid.

Further improvement of the CS-based imaging system can be achieved, based on:

(i) The application of optimization techniques to find the position of the transducers that maximizes the quality of CS images, as completed in [29,31] for a millimeter-wave security screening system.
(ii) The integration of the Fourier-based imaging into the CS algorithm. This study has been undertaken due to the requirement of evenly spaced samples for FFTs. An alternative could be to introduce Non-Uniform FFT (NUFFT), but its capability to filter out non-desired contributions using a reduced set of samples will have to be evaluated.

## 6. Conclusions

This contribution has evaluated the feasibility of applying CS techniques to reduce the number of samples required for ultrasound imaging. More concisely, the main goal is to evaluate how far the number of elements of an array of transducers can be decreased. From the presented results, and considering the trade-off between the maximum CS performance and the technical restrictions that prevents a full random sampling scheme, it has been concluded that accurate imaging can be achieved with 40% of the number of transducers required by a full array.
